# Educational inequalities in cervical cancer mortality in the Baltic countries and Finland in the context of organized screening: A register‐based study 2000–2015

**DOI:** 10.1002/ijc.70339

**Published:** 2026-01-24

**Authors:** Oskar Nõmm, Kaire Innos, Domantas Jasilionis, Juris Krumins, Pekka Martikainen, Kersti Pärna, Andrew Stickley, Mall Leinsalu

**Affiliations:** ^1^ Department of Epidemiology and Biostatistics National Institute for Health Development Tallinn Estonia; ^2^ Institute of Family Medicine and Public Health University of Tartu Tartu Estonia; ^3^ Laboratory of Demographic Data Max Planck Institute for Demographic Research Rostock Germany; ^4^ Demographic Research Centre Vytautas Magnus University Kaunas Lithuania; ^5^ Demography Unit, Faculty of Business, Management and Economics University of Latvia Riga Latvia; ^6^ Helsinki Institute for Demography and Population Health, Faculty of Social Sciences University of Helsinki Helsinki Finland; ^7^ Max Planck—University of Helsinki Center for Social Inequalities in Population Health, Faculty of Social Sciences, University of Helsinki Helsinki Finland; ^8^ Laboratory of Population Health Max Planck Institute for Demographic Research Rostock Germany; ^9^ Stockholm Centre for Health and Social Change Södertörn University Huddinge Sweden

**Keywords:** cervical cancer, education, inequalities, mortality, screening

## Abstract

Cervical cancer (CC) mortality in the Baltic countries remains high. We explored CC mortality trends and educational inequalities in CC mortality in the Baltics in the context of organized CC screening (introduced in 2004 in Lithuania, 2006 in Estonia and 2009 in Latvia) and compared the results with Finland (where screening started in 1963). Data for the Baltic countries came from longitudinal mortality follow‐up studies of population censuses in 2000/2001 and 2011, and data for Finland from the longitudinal register‐based population data file of Statistics Finland. CC deaths (ICD‐10 code C53) were linked from national mortality registries. Information on education was census‐ or register‐based. Overall and education‐specific age‐standardized mortality rates (ASMRs) and mortality rate ratios were calculated for 2000–2007 and 2008–2015 for women aged 30–49 and 50–64 years. The Baltic countries had 5–9 times higher overall ASMRs than Finland and much larger inequalities in CC mortality between low‐ and highly educated women. From 2000–2007 to 2008–2015 absolute inequalities in younger women reduced in all countries, except Latvia and relative inequalities increased in Estonia and Latvia. In older women, absolute inequalities increased in the Baltics but not in Finland; relative inequalities increased in all countries. The reduction in CC mortality and in absolute inequalities in younger women in Estonia and Lithuania may be associated with the introduction of organized screening. However, increasing CC mortality among older low‐educated women in the Baltic countries is alarming, indicating that they have not benefitted equally from CC prevention.

## Graphical abstract

What's new?

This study shows that absolute and relative educational inequalities in cervical cancer mortality are much larger in the Baltic countries than in Finland, where an organized screening programme was introduced more than 40 years earlier. After the introduction of organized screening, cervical cancer mortality declined among low‐educated women aged 30–49 years in Estonia and Lithuania, but increased among low‐educated women aged 50–64 years in all Baltic countries. Compared to their counterparts, older low‐educated women in the Baltic countries have not benefitted equally from cervical cancer prevention initiatives, calling for new and innovative approaches in these countries.

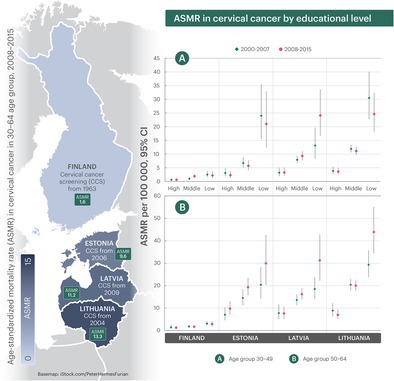

**Abbreviations**
ASMR: age‐standardized mortality rate
CC: cervical cancer
CI: confidence interval
EU: European Union
GP: general practitioner
HPV: human papillomavirus
ICD: International classification of diseases
ISCED: International standard classification of education
OECD: Organization for Economic Co‐Operation and Development
RR: rate ratio

## 1 INTRODUCTION

Cervical cancer (CC) is the fourth leading cause of cancer mortality among women globally, with an estimated 348,000 deaths in 2022, of which the vast majority occurred in developing countries.^1^ Over the past few decades, both CC incidence and mortality rates have declined in many areas of the world. According to the Global Burden of Disease study, the overall global age‐standardized CC incidence rate declined from 14.91 to 13.35 per 100,000 between 1990 and 2019, while the mortality rate reduced from 8.48 to 6.51 per 100,000 during the same period.^2^ The decline in CC incidence and mortality, mainly attributable to effective population‐based screening, has been larger in high human development countries that started organized screening programs earlier.^2^, ^3^, ^4^ CC is now considered highly preventable through interventions such as vaccination against its main risk factor, human papillomavirus (HPV),^5^ and screening for precancerous cervical lesions.^3^

Accumulating evidence suggests that CC risk and progression are strongly influenced by socioeconomic status.^6^, ^7^, ^8^, ^9^ Higher CC incidence and mortality have been reported in populations characterized by high levels of deprivation and low levels of socioeconomic and human development.^9^, ^10^, ^11^ Screening participation, vaccination against HPV, and factors such as earlier age at first intercourse, multiple sexual partners, multiparity, or smoking, which affect CC risk and progression, are strongly influenced by socioeconomic status.^6^ There is some indication that socioeconomic inequalities in CC screening attendance are smaller in countries with organized screening compared to more opportunistic screening.^12^, ^13^ Organized screening may thus reduce inequalities in CC by reaching vulnerable groups that otherwise use health services less frequently. A recent study of 18 European countries showed that low‐educated women had three times higher CC mortality compared to their highly educated counterparts.^14^

There are several reasons to study CC in the Baltic countries. Together with some Eastern European countries, the Baltic countries have the highest overall CC incidence and mortality in Europe,^1^, ^15^ which has been ascribed to the high prevalence of high‐risk HPV types, late‐stage diagnosis and suboptimal screening and vaccination coverage.^16^ Indeed, organized population‐based CC screening was introduced as late as 2004 in Lithuania, 2006 in Estonia and 2009 in Latvia. Before that, screening was opportunistic and depended on women's visits to gynecologists. All programs in the Baltic countries were Pap‐smear based but had structural differences and lacked comprehensive quality assurance. In Estonia, women aged 30–55 years were invited to screening every 5 years using printed and electronic letters; in Latvia, women aged 25–69 years were invited every 3 years using printed letters; and in Lithuania, 29–59‐year‐old women were invited every 3 years mainly through a verbal invitation from primary practitioners.^16^ During the period between 1990 and 2018, the CC incidence began to decline from 2006 onwards in Lithuania, with the annual percentage change (APC) being −3.5%; from 2013 in Estonia (APC = −7.1%); and from 2014 in Latvia (APC = −4.3%),^16^ that may indicate some beneficial impact of organized screening. There is also evidence that socioeconomic inequalities in CC mortality are particularly high in the Baltic and Eastern European countries where more than half of total CC mortality is associated with having a less‐than‐high educational level.^14^

This study aims to explore trends and educational inequalities in CC mortality in Estonia, Latvia, and Lithuania in the context of the introduction of organized CC screening programs and compare the results with Finland. The focus on mortality is warranted as it is an ultimate endpoint when assessing the progress of CC prevention, combining advances in primary prevention, early detection, and cancer care. Two periods will be compared: 2000–2007, when CC screening in the Baltic countries was largely non‐existent or only partially functioning,^17^ and 2008–2015, by the end of which CC screening had been ongoing for almost a decade. Finland is a neighboring country where population‐based CC screening was initiated as early as in 1963, targeting women aged 30–60 years every 5 years.^18^ Although CC incidence in Finland was already low before the start of organized screening, both incidence and mortality rates declined by more than 40% in the decade after the start of CC screening.^19^ According to 2018 estimates, CC mortality in Finland is one of the lowest in Europe.^20^

## 2 MATERIALS AND METHODS

### 2.1 Data

Data for Estonia, Latvia, and Lithuania were obtained from longitudinal mortality follow‐up studies of population censuses in 2000 (2001 in Lithuania) and 2011 involving all permanent residents. The censuses in the Baltic countries combined traditional survey‐based enumeration (the share of coverage ranged from 91% in Latvia to 98% in Estonia) and register‐based enumeration.^21^ The register‐only‐based data did not include information about socioeconomic status and were therefore excluded from the analysis. All individuals were followed from the census date until the date of death or emigration or until the end of the follow‐up period. The date and cause of death were linked from national mortality registries. All data linkages were performed by National Statistical Offices. Corresponding data for Finland were obtained from the longitudinal register‐based population data file of Statistics Finland covering the total population. Population exposures were estimated by adding up the number of person‐years lived by individuals within each 5‐year interval age group in a given period. Deaths were allocated to age intervals using the age at death. Data were anonymized and aggregated into multidimensional frequency tables combining deaths and population exposures split by four study periods and sociodemographic variables before they were delivered for research purposes. To address our research questions, we further organized data into two sub‐periods: 2000–2007 and 2008–2015. This study focuses on women aged 30–64 years, roughly corresponding to the age inclusion criteria in national CC screening programs. We further divided our analytical sample into 30–49‐ and 50–64‐year age groups to distinguish women at reproductive and menopausal age as this may relate to differences in the regularity of visiting gynecologists, thus also potentially affecting early cancer detection.

### 2.2 Measures

Deaths from cervical cancer were defined as C53 using the 10th revision of the International Classification of Diseases (ICD‐10). Sociodemographic data are census‐based and were harmonized following the standard study protocol. Educational level was categorized using the International Standard Classification of Education (ISCED) 2011 as high (ISCED categories 5–8), middle (3, 4), or low (1, 2).^22^ The percentage of missing data for education was less than 1% in all instances and these data were excluded from the analysis.

### 2.3 Statistical analysis

To examine the trends and inequalities in CC mortality, age‐standardized mortality rates (ASMRs) per 100,000 person‐years and mortality rate ratios (RR) together with 95% confidence intervals (CI) were calculated for both periods and both age groups using the European Standard Population.^23^ Differences in ASMRs were used to assess absolute inequalities, and RRs to assess relative inequalities in CC mortality between educational groups. Statistical analyses were performed using STATA 17.0 (Stata Corp., College Station, Texas, USA).

## 3 RESULTS

**FIGURE 1:**
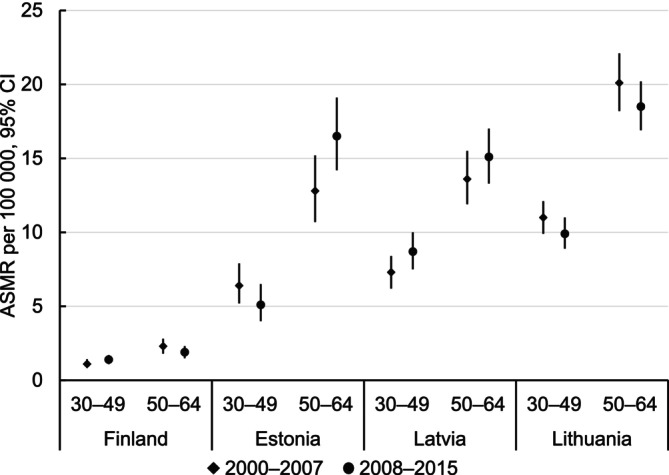
Age‐standardized cervical cancer mortality in Finland, Estonia, Latvia and Lithuania, 2000–2015. ASMR, Age‐standardized (European) mortality rate per 100,000 person years; CI, confidence interval.

This study included about 45 million person‐years and 3345 CC deaths ranging from 321 in Finland to 1683 in Lithuania among 30–64‐year‐old women (Table 1). The percentage of highly educated women increased in all countries and in both age groups over the study period. The proportion of low‐educated women diminished in all countries among 50–64‐year‐olds, and in Finland also among 30–49‐year‐olds, while it remained considerably lower in the Baltic countries than in Finland in both age groups. The overall ASMRs per 100,000 person years for CC were 5–9 times higher in the Baltic countries compared to Finland (Supplementary Table 1). In 2000–2007, ASMRs ranged from 1.6 in Finland to 14.5 in Lithuania. Between 2000–2007 and 2008–2015, ASMRs remained the same in Finland, declined slightly in Lithuania, and increased in Estonia and Latvia. An age‐specific analysis revealed similarly large CC mortality gaps between the Baltic countries and Finland (Figure 1, Supplementary Table 1). Between 2000–2007 and 2008–2015, ASMRs among 30–49‐year‐old women increased in Finland and Latvia but decreased in Estonia and Lithuania. Among 50–64‐year‐olds, ASMRs increased in Estonia and Latvia but decreased in Finland and Lithuania. Both absolute and relative differences in ASMRs between the Baltic countries and Finland diminished somewhat among 30–49‐year‐olds (except for the absolute difference for Latvia) but increased among 50–64‐year‐olds (except for the absolute difference for Lithuania), calculated from Supplementary Table 1.

**TABLE 1:** Characteristics of the study populations, women aged 30–64 years in Finland, Estonia, Latvia and Lithuania, 2000–2015.

Country	Period			Age group 30–49			Age group 50–64		
		Total		Educational level			Educational level		
		Deaths	Person‐years	High	Middle	Low	High	Middle	Low
		*N*	*N*	%	%	%	%	%	%
Finland	2000–2007	161	9,873,655	41.5	42.5	16.0	26.4	34.9	38.7
	2008–2015	160	9,821,810	44.0	43.6	12.4	35.1	40.9	24.0
Estonia	2000–2007	230	2,507,051	40.9	51.9	7.2	34.3	44.7	21.0
	2008–2015	259	2,517,900	42.4	48.6	9.0	42.2	47.0	10.8
Latvia	2000–2007	406	4,017,624	22.3	69.7	8.0	18.5	57.6	23.9
	2008–2015	446	3,843,069	30.0	60.4	9.6	26.3	64.2	9.5
Lithuania	2001–2007	824	5,710,308	22.2	72.1	5.7	19.3	57.5	23.2
	2008–2015	859	6,231,460	28.2	62.2	9.6	24.6	66.9	8.5

**FIGURE 2:**
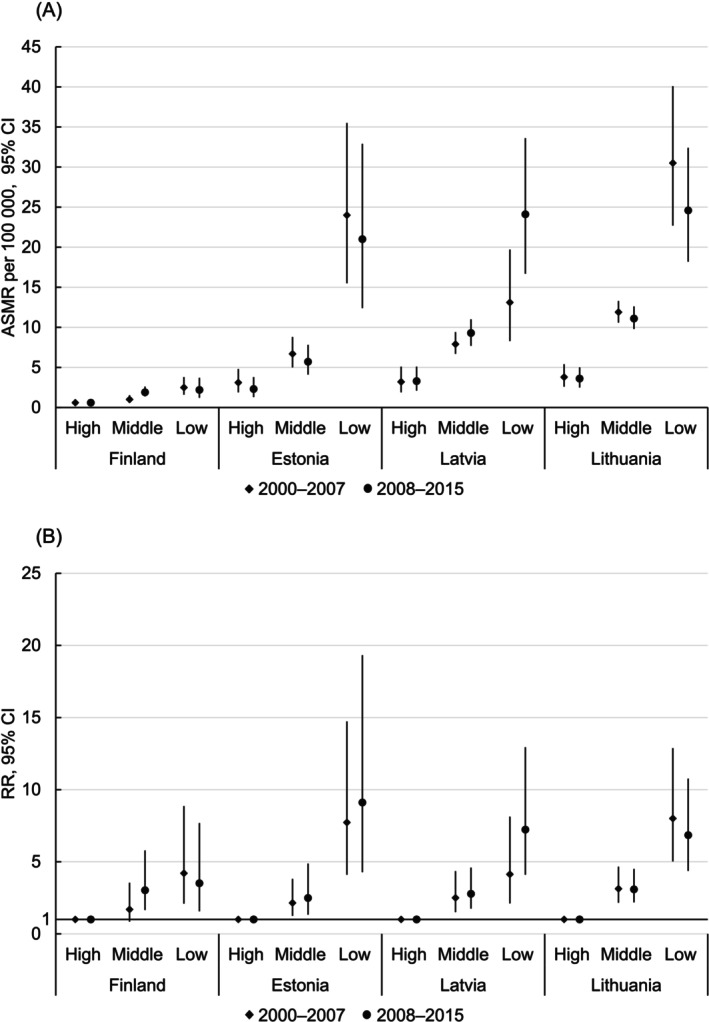
Absolute (A) and relative (B) inequalities in cervical cancer mortality by educational level among women aged 30–49 years in Finland, Estonia, Latvia, and Lithuania, 2000–2015. ASMR, age‐standardized (European) mortality rate per 100,000 person years; RR, mortality rate ratio; CI, confidence interval.

**FIGURE 3:**
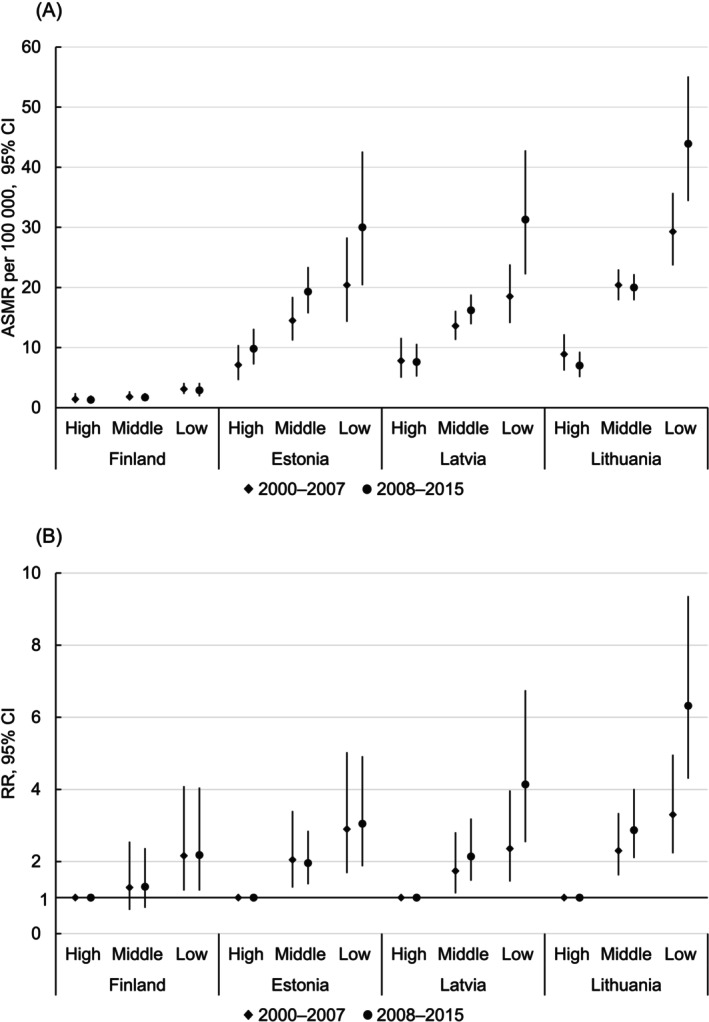
Absolute (A) and relative (B) inequalities in cervical cancer mortality by educational level among women aged 50–64 years in Finland, Estonia, Latvia, and Lithuania, 2000–2015. ASMR, age‐standardized (European) mortality rate per 100,000 person years; RR, mortality rate ratio; CI, confidence interval.

Large educational inequalities in CC mortality were observed in all countries (Figures 2 and 3; Supplementary Tables 2 and 3). In nearly all instances, both absolute and relative inequalities were considerably larger in the Baltic countries than in Finland. Remarkably, ASMRs for low‐educated women in Finland remained lower than for highly educated women in the Baltic countries.

Among 30–49‐year‐old women (Figure 2, Supplementary Table 2), in 2000–2007, ASMRs were lowest in Finland and highest in Lithuania, ranging respectively from 0.6 to 3.8 for highly educated, from 1.0 to 11.9 for middle‐educated, and from 2.5 to 30.5 for low‐educated women. Compared to highly educated, RRs for middle‐educated women ranged from 1.70 in Finland to 3.13 in Lithuania; for low‐educated women, RRs ranged from 4.13 in Latvia to 8.01 in Lithuania, but were also high in Estonia (7.73). Between 2000–2007 and 2008–2015, ASMRs declined in all educational groups in Estonia and Lithuania and among low‐educated women in Finland; ASMRs increased in all educational groups in Latvia and among middle‐educated women in Finland. As a result, absolute educational inequalities in CC mortality reduced in all countries except Latvia and among middle‐educated women in Finland. Compared to highly educated, RRs for middle‐educated women increased in all countries except Lithuania; for low‐educated, RRs increased in Estonia and Latvia.

Among 50–64‐year‐old women (Figure 3, Supplementary Table 3), similar to the younger age group, in 2000–2007 ASMRs were lowest in Finland and highest in Lithuania, ranging respectively from 1.4 to 8.9 for highly educated, from 1.8 to 20.4 for middle‐educated, and from 3.1 to 29.2 for low‐educated. Compared to highly educated, RRs for middle‐educated women ranged from 1.28 in Finland to 2.30 in Lithuania; for low‐educated women, RRs ranged from 2.16 in Finland to 3.30 in Lithuania. Between 2000–2007 and 2008–2015, ASMRs declined in all educational groups in Finland, among highly educated women in Latvia, and among highly and middle‐educated women in Lithuania. ASMRs increased in all educational groups in Estonia, among low‐ and middle‐educated women in Latvia, and among low‐educated women in Lithuania. Absolute educational inequalities in CC mortality increased considerably in all Baltic countries but not in Finland. Compared to highly educated, RRs for middle‐educated women increased in all countries except Estonia; for low‐educated women, they increased in all countries.

## 4 DISCUSSION

Overall CC mortality was 5–9 times higher in the Baltic countries than in Finland. In nearly all instances, both absolute and relative educational inequalities in CC mortality were much larger in the Baltic countries, particularly between low‐ and highly educated women. Between 2000–2007 and 2008–2015, among 30–49‐year‐olds, the absolute decline in CC mortality was larger among the low‐educated in all countries except Latvia, where ASMRs increased in all educational groups. In Finland, the ASMR increased among middle‐educated women. Among 50–64‐year‐olds, the absolute increase was far largest among low‐educated women in all Baltic countries, but ASMRs increased also among high and middle‐educated women in Estonia and among middle‐educated women in Latvia. In Finland, the ASMRs declined in all educational groups. As a consequence of these education specific trends, absolute inequalities in CC mortality among 30–49‐year‐old women reduced in all countries except Latvia and for the middle‐educated in Finland; while relative inequalities increased in Estonia, Latvia, and for middle‐educated women in Finland. Among 50–64‐year‐olds, absolute inequalities increased considerably in all Baltic countries but not in Finland, while relative inequalities increased in all countries except among middle‐educated women in Estonia.

### 4.1 Strengths and limitations

The major strength of this study is the use of highly comparable longitudinal register‐based data with reliable individual‐level information on sociodemographic characteristics and CC deaths from four countries to examine trends and inequalities in CC mortality over a 15‐year period. However, the study also has some limitations. First, the exclusion of register‐only‐based data from censuses in the Baltic countries could be a potential source of bias if these data systematically differed from survey‐based enumeration data. The results from a sensitivity analysis showed that by excluding register‐only‐based data we have slightly underestimated overall CC mortality in Latvia, although the differences in ASMRs were not statistically significant (Supplementary Table 4). Even though we cannot exclude the possibility that the effects may have differed by educational level, we believe that this bias was unlikely to have had any major impact on our conclusions related to CC mortality and its changes in Latvia. Second, educational level was the only indicator of socioeconomic status we could access, and it is possible that the results would have differed if we had used income or occupational status instead. Third, although the relatively small number of CC deaths resulted in large CIs for some of the estimates thus hampering the comparisons, we believe that our results are robust in that they reflect the true pattern regarding the trends and educational inequalities in CC mortality between and within countries. Fourth, we only had access to education‐stratified mortality data, and using both incidence and mortality data may have revealed different results regarding the trends in inequalities in CC in the context of organized screening. Finally, our study covered the period from 2000 to 2015 and it is possible that recent developments in CC screening programs have impacted educational inequalities in CC mortality differently from our findings; a fruitful target for future studies.

### 4.2 Interpretation of the findings

The huge differences in CC mortality between the Baltic countries and Finland corroborate earlier findings^15^, ^20^ and reflect historical differences in CC incidence and mortality, late implementation of organized screening in the Baltic countries, as well as disparities in cancer care and survival.^4^, ^17^, ^24^, ^25^ From 2000–2007 to 2008–2015, overall CC mortality increased in Estonia and Latvia but decreased in Lithuania. CC screening in the Baltic countries has been characterized by its opportunistic nature, low attendance and lack of sufficient quality assurance.^16^ Although the attendance rate has increased over the years, it was still relatively low as late as in 2018/2019, being 40% in Latvia, 46% in Estonia and 54% in Lithuania.^16^ In Finland, the attendance rate was 72% in 2022, which was one of the highest in the European region.^26^ Until recently, CC screening participation in Lithuania depended heavily on the frequency of GP (general practitioner) visits, as GP referral was a prerequisite for screening attendance,^27^ while in Latvia, only women with a physical invitation letter were able to participate.^28^ In Estonia, women without health insurance were not included in organized screening until 2021.^29^ Despite the overall high coverage of cervical cytology in Estonia,^30^ women were shown to receive a CC diagnosis regardless of several prior Pap‐smears^31^ and previous studies have highlighted the suboptimal performance of cervical cytology practices.^32^ Estonian women with gaps in insurance coverage were shown to have 23% higher CC incidence compared to those with consistent coverage.^33^ Other factors that may have contributed to high overall CC mortality in the Baltic countries are the high HPV prevalence in the general population,^16^ but also a survival deficit due to insufficient early diagnosis and inadequate treatment. In 2007–2018, 55.6% of CC cases in Estonia were diagnosed at an advanced stage (TNM stages II–IV); between 2007–2009 and 2015–2018, this proportion increased from 51% to 58%.^34^ From 2005–2009 to 2014–2015, CC stage distribution also shifted toward advanced stages in Lithuania, while in Latvia the proportion of stage I increased.^16^ According to the EUROCARE 5 study, the 5‐year relative survival in 2000–2007 was 67% in Finland, 64% in Estonia, 56% in Lithuania and 51% in Latvia.^25^ In 2010–2014, the 5‐year relative survival was 67% in Finland and Estonia, 59% in Lithuania and 56% in Latvia.^35^ The substantial lag in economic development in the Baltic countries has contributed to shortages in healthcare resources affecting cancer care. A recent Organization for Economic Co‐Operation and Development (OECD) Country Cancer Profiles publication highlighted healthcare staff and system shortages in all of the Baltic countries, with fewer nurses and physicians relative to new cancer cases and a lower availability of radiotherapy equipment compared to the European Union (EU) average.^36^ According to the OECD, there are long waiting times for cancer care delivery in Latvia and Lithuania, financial barriers such as co‐payments for inpatient care and cancer drug prescriptions in Latvia,^27^, ^28^ whereas Estonia ranks highest in the EU in terms of reporting unmet needs for overall medical care.^29^ In Latvia and Lithuania, the direction of the change in CC mortality was similar in both the 30–49 and 50–64 age groups, while in Estonia the increase was mainly due to a considerable CC mortality increase in older women. Although the slight decline in CC mortality in Lithuania and among younger women in Estonia might reflect the first signs of the effect of CC screening, the results generally indicate inefficiencies in CC screening programs in the Baltics. It is possible that the lower upper‐age limit (i.e., age 55) for CC screening in Estonia may have contributed to the substantial CC mortality increase among 50–64‐year‐olds in this setting. This is an important obstacle to achieving effective screening as older women are also more likely to miss opportunistic screening due to the reduced regularity of visiting a gynecologist for checkups and underestimating their own risk of having gynecological conditions. Consequently, older women in Estonia have an increased risk of being diagnosed with more advanced‐stage CC.^34^

Our finding that lower educational level was consistently associated with higher CC mortality and that both absolute and relative educational inequalities were considerably larger in the Baltic countries than in Finland strongly align with previous research.^14^ It should be noted that the proportion of low‐educated women, particularly in the 50–64 age group, was larger in Finland than in the Baltic countries. Thus, the low‐educated group in the Baltic countries might be more affected by health selection, resulting in a higher concentration of women with elevated CC risk factors. This compositional difference may partly explain the larger educational gap in CC mortality observed in the Baltics. The association between education and CC mortality can be explained by the impact of its main risk factors, HPV infection and non‐attendance in CC screening programs^11^ that can influence the development of invasive cancer. Early age at first sexual intercourse, the number of lifetime sexual partners, and the partner's extramarital sexual relations can increase the likelihood of HPV infection and are more prevalent or remain poorly acknowledged among lower‐educated women.^6^, ^37^ The potential effects of these risk behaviors can be mitigated through organized CC screening, although attendance is also less frequent among lower‐educated women. In Estonia, lower educational level has been associated with poorer uptake of Pap‐smears,^38^ higher CC risk,^33^ and with a more advanced stage at CC diagnosis.^34^ Similar associations have been reported for Lithuania where lower education has been linked to non‐attendance of CC screening,^39^ increased CC risk,^40^ and lower survival.^41^, ^42^ There is some evidence that social inequalities may also affect receipt of cancer treatment. Research has shown that in Estonia, low‐educated breast cancer patients receive less radiotherapy, which may also apply to CC.^43^ Other behavioral risk factors affecting screening attendance, such as smoking and being overweight or obese, are not only highly prevalent in the Baltic countries,^36^, ^38^, ^39^ but the prevalence is substantially higher among low‐educated women.^44^ Between 2000–2007 and 2008–2015, among 30–49‐year‐old women, the absolute decline in CC mortality was larger among the low‐educated in all countries except Latvia, leading to reduced absolute educational inequalities in CC mortality. In contrast, among 50–64‐year‐old women, absolute educational inequalities in CC mortality increased as mortality rates increased substantially more among the low‐educated in all Baltic countries. Thus, the results indicate that organized screening may have contributed to reducing educational inequalities in CC mortality in the Baltic countries, at least on an absolute scale and in younger women who are better covered by organized screening. A promising new approach to ensure more equitable screening coverage is HPV self‐sampling as an alternative option for participating in CC screening, which has been found to increase screening attendance in Estonia, particularly in older screening cohorts.^45^

## 5 CONCLUSIONS

The results suggest that the delayed introduction of organized CC screening in the Baltic countries accompanied by low attendance, a high prevalence of opportunistic screening and poor quality assurance, together with gaps in primary prevention and cancer care may explain not only the higher overall CC mortality in these countries but also the considerably larger educational inequalities. The reduction in CC mortality and educational inequalities in 30–49‐year‐old women in Estonia and Lithuania may be associated with the introduction of organized screening. However, increasing CC mortality among 50–64‐year‐old low‐educated women in the Baltics is alarming, indicating that they have not benefitted equally from CC prevention. Therefore, new and innovative approaches to equitable prevention are needed to further reduce CC mortality in these countries.

## AUTHOR CONTRIBUTIONS

**Oskar Nõmm:** Writing – original draft; writing – review and editing; formal analysis. **Kaire Innos:** Conceptualization; writing – review and editing. **Domantas Jasilionis:** Data curation; writing – review and editing. **Juris Krumins:** Data curation; writing – review and editing. **Pekka Martikainen:** Data curation; writing – review and editing. **Kersti Pärna:** Writing – review and editing. **Andrew Stickley:** Writing – review and editing. **Mall Leinsalu:** Conceptualization; funding acquisition; writing – original draft; writing – review and editing; formal analysis; project administration; data curation; supervision.

## FUNDING INFORMATION

The data collection for this study was financed by *Riksbankens Jubileumsfond*—The Swedish Foundation for Humanities and Social Sciences (grant P15‐0520:1). The work by Oskar Nõmm, Kaire Innos, and Mall Leinsalu was supported by the Estonian Research Council (grant No PRG722 and PRG2543). Pekka Martikainen was supported by the European Research Council under the European Union's Horizon 2020 research and innovation programme (grant agreement No 101019329), the Strategic Research Council (SRC) within the Research Council of Finland grants for ACElife (#352543–352572) and LIFECON (# 345219), the Research Council of Finland profiling grant for SWAN and FooDrug, and grants to the Max Planck—University of Helsinki Center from the Jane and Aatos Erkko Foundation (#210046), the Max Planck Society (#5714240218), University of Helsinki (#77204227), and Cities of Helsinki, Vantaa, and Espoo. Juris Krumins was supported by the Latvian Council of Science project “DemoMig”.

## CONFLICT OF INTEREST STATEMENT

The authors declare that they have no competing interests.

## Supporting information


**Data S1:** Supplementary Tables

## Data Availability

Aggregated age‐specific data that are minimally required to reproduce the findings of this study are available from the corresponding author upon reasonable request.

## References

[1] Bray F , Laversanne M , Sung H , et al. Global cancer statistics 2022: GLOBOCAN estimates of incidence and mortality worldwide for 36 cancers in 185 countries. CA Cancer J Clin. 2024;74:229‐263. doi:10.3322/caac.21834 38572751

[2] Zhang X , Zeng Q , Cai W , Ruan W . Trends of cervical cancer at global, regional, and national level: data from the global burden of disease study 2019. BMC Public Health. 2021;21:894. doi:10.1186/s12889-021-10907-5 33975583 PMC8114503

[3] Vaccarella S , Lortet‐Tieulent J , Plummer M , Franceschi S , Bray F . Worldwide trends in cervical cancer incidence: impact of screening against changes in disease risk factors. Eur J Cancer. 2013;49:3262‐3273. doi:10.1016/j.ejca.2013.04.024 23751569

[4] Vaccarella S , Franceschi S , Engholm G , Lönnberg S , Khan S , Bray F . 50 years of screening in the Nordic countries: quantifying the effects on cervical cancer incidence. Br J Cancer. 2014;111:965‐969. doi:10.1038/bjc.2014.362 24992581 PMC4150271

[5] de Martel C , Plummer M , Vignat J , Franceschi S . Worldwide burden of cancer attributable to HPV by site, country and HPV type. Int J Cancer. 2017;141:664‐670. doi:10.1002/ijc.30716 28369882 PMC5520228

[6] Franceschi S , Plummer M , Clifford G , et al. Differences in the risk of cervical cancer and human papillomavirus infection by education level. Br J Cancer. 2009;101:865‐870. doi:10.1038/sj.bjc.6605224 19654578 PMC2736843

[7] Donkers H , Bekkers R , Galaal K . Systematic review on socioeconomic deprivation and cervical cancer: inequalities in survival. J Health Care Poor Underserved. 2021;32:751‐766. doi:10.1353/hpu.2021.0103 34120975

[8] Eslahi M , Pizzato M , Heikkinen S , et al. Socioeconomic position and risk of cervical cancer in the Nordic countries: results from the Nordic occupational cancer study. Int J Cancer. 2025;157:95‐102. doi:10.1002/ijc.35349 40079673

[9] Fay M , Hu M , Hajizadeh M . Socioeconomic inequalities in cervical cancer mortality in Canada, 1990 and 2019: a trend analysis. Public Health. 2024;227:210‐218. doi:10.1016/j.puhe.2023.12.014 38241902

[10] Singh GK , Azuine RE , Siahpush M . Global inequalities in cervical cancer incidence and mortality are linked to deprivation, low socioeconomic status, and human development. Int J MCH AIDS. 2012;1:17‐30. doi:10.21106/ijma.12 27621956 PMC4948158

[11] Parikh S , Brennan P , Boffetta P . Meta‐analysis of social inequality and the risk of cervical cancer. Int J Cancer. 2003;105:687‐691. doi:10.1002/ijc.11141 12740919

[12] de Prez V , Jolidon V , Willems B , Cullati S , Burton‐Jeangros C , Bracke P . Cervical cancer screening programs and their context‐dependent effect on inequalities in screening uptake: a dynamic interplay between public health policy and welfare state redistribution. Int J Equity Health. 2021;20:211. doi:10.1186/s12939-021-01548-6 34560888 PMC8464130

[13] Palència L , Espelt A , Rodríguez‐Sanz M , et al. Socio‐economic inequalities in breast and cervical cancer screening practices in Europe: influence of the type of screening program. Int J Epidemiol. 2010;39:757‐765. doi:10.1093/ije/dyq003 20176587

[14] Vaccarella S , Georges D , Bray F , et al. Socioeconomic inequalities in cancer mortality between and within countries in Europe: a population‐based study. Lancet Reg Health Eur. 2023;25:100551. doi:10.1016/J.LANEPE.2022.100551 36818237 PMC9929598

[15] Arbyn M , Antoine J , Mägi M , et al. Trends in cervical cancer incidence and mortality in the Baltic countries, Bulgaria and Romania. Int J Cancer. 2011;128:1899‐1907. doi:10.1002/ijc.25525 20568103

[16] Kojalo U , Tisler A , Pärna K , et al. An overview of cervical cancer epidemiology and prevention in the Baltic States. BMC Public Health. 2023;23:660. doi:10.1186/s12889-023-15524-y 37029357 PMC10080753

[17] Vaccarella S , Franceschi S , Zaridze D , et al. Preventable fractions of cervical cancer via effective screening in six Baltic, central, and eastern European countries 2017–40: a population‐based study. Lancet Oncol. 2016;17:1445‐1452. doi:10.1016/S1470-2045(16)30275-3 27567054 PMC5052457

[18] Anttila A , Nieminen P . Cervical cancer screening programme in Finland. Eur J Cancer. 2000;36:2209‐2214. doi:10.1016/s0959-8049(00)00311-7 11072206

[19] Larønningen S , Arvidsson G , Bray F , et al. NORDCAN: Cancer Incidence, Mortality, Prevalence and Survival in the Nordic Countries, Version 9.5 (19.06.2025). Association of the Nordic Cancer Registries. Cancer Registry of Norway. Available from: https://nordcan.iarc.fr/ Accessed November 11, 2025.

[20] Arbyn M , Weiderpass E , Bruni L , et al. Estimates of incidence and mortality of cervical cancer in 2018: a worldwide analysis. Lancet Glob Health. 2020;8:e191‐e203. doi:10.1016/S2214-109X(19)30482-6 31812369 PMC7025157

[21] Statistical Office of Estonia, Central Statistical Bureau of Latvia, Statistics Lithuania . 2011 population and housing censuses in Estonia, Latvia and Lithuania. 2015.

[22] UNESCO . International Standard Classification of Education ISCED 2011. UNESCO Institute of Statistics; 2012.

[23] Waterhouse J , Muir C , Correa P , Powell J . Cancer Incidence in Five Continents. Vol 3. IARC Scientific Publications; 1976:584.

[24] Aareleid T , Thomson H , Pukkala E , Hakama M . Cervical cancer incidence and mortality trends in Finland and Estonia: a screened vs. an unscreened population. Eur J Cancer. 1993;29:745‐749. doi:10.1016/s0959-8049(05)80359-4 8471334

[25] Sant M , Chirlaque Lopez MD , Agresti R , et al. Survival of women with cancers of breast and genital organs in Europe 1999‐2007: results of the EUROCARE‐5 study. Eur J Cancer. 2015;51:2191‐2205. doi:10.1016/j.ejca.2015.07.022 26421822

[26] OECD/European Commission . EU Country Cancer Profile: Finland 2025, EU Country Cancer Profiles. OECD Publishing; 2025. doi:10.1787/1b14100d-en

[27] OECD/European Commission . EU Country Cancer Profile: Lithuania 2025, EU Country Cancer Profiles. OECD Publishing; 2025. doi:10.1787/b260e7d1-en

[28] OECD/European Commission . EU Country Cancer Profile: Latvia 2025, EU Country Cancer Profiles. OECD Publishing; 2025. doi:10.1787/f23ce73c-en

[29] OECD/European Commission . EU Country Cancer Profile: Estonia 2025, EU Country Cancer Profiles. OECD Publishing; 2025. doi:10.1787/bb4eec73-en

[30] Partanen V , Antilla A , Heinävaara S , et al. NordScreen: Performance indicators on cancer screening in the Nordic countries, Version 1.1 (15.5.2019) [online]. eHealth Core Facility, Department of Laboratory Medicine, Karolinska Institutet. http://www.nordscreen.org accessed 20 March 2025.

[31] Orumaa M , Innos K , Suurna M , Veerus P . Cervical cancer screening history among women diagnosed with cervical cancer in Estonia 2017–18. Eur J Public Health. 2023;33:64‐68. doi:10.1093/eurpub/ckac176 36469798 PMC9898000

[32] Orumaa M , Innos K , Suurna M , Salumäe L , Veerus P . Quality assessment of cervical cytology practices in Estonia from 2007 to 2018. Cancer Control. 2022;29. doi:10.1177/10732748221141794 PMC9793007 36542780

[33] Nõmm O , Veerus P , Orumaa M , Innos K . Effect of pap‐smear and sociodemographic factors on cervical cancer risk in Estonia: a population‐based case‐control study. Cancer Epidemiol. 2022;80:102231. doi:10.1016/j.canep.2022.102231 35901623

[34] Šavrova A , Jaal J , Nõmm O , Innos K . Factors associated with advanced‐stage diagnosis of cervical cancer in Estonia: a population‐based study. Public Health. 2023;225:369‐375. doi:10.1016/J.PUHE.2023.10.025 37989009

[35] Allemani C , Matsuda T , Di Carlo V , et al. Global surveillance of trends in cancer survival 2000‐14 (CONCORD‐3): analysis of individual records for 37 513 025 patients diagnosed with one of 18 cancers from 322 population‐based registries in 71 countries. Lancet. 2018;391:1023‐1075. doi:10.1016/S0140-6736(17)33326-3 29395269 PMC5879496

[36] OECD/European Commission . EU Country Cancer Profiles Synthesis Report 2025, EU Country Cancer Profiles. OECD Publishing; 2025. doi:10.1787/20ef03e1-en

[37] Kliucinskas M , Nadisauskiene RJ , Minkauskiene M . Prevalence and risk factors of HPV infection among high‐risk rural and urban Lithuanian women. Gynecol Obstet Invest. 2006;62:173‐180. doi:10.1159/000093572 16717474

[38] Suurna M , Orumaa M , Ringmets I , Pärna K . Inequalities in reported use of cervical screening in Estonia: results from cross‐sectional studies in 2004–2020. BMC Womens Health. 2022;22:545. doi:10.1186/s12905-022-02123-z 36566176 PMC9789641

[39] Petkeviciene J , Ivanauskiene R , Klumbiene J . Sociodemographic and lifestyle determinants of non‐attendance for cervical cancer screening in Lithuania, 2006–2014. Public Health. 2018;156:79‐86. doi:10.1016/j.puhe.2017.12.014 29408192

[40] Smailytė G , Jasilionis D , Vincerzevskiene I , et al. Educational differences in incidence of cancer in Lithuania, 2001–2009: evidence from census‐linked cancer registry data. Eur J Cancer Prev. 2015;24:261‐266. doi:10.1097/CEJ.0000000000000036 24743349

[41] Smailytė G , Jasilionis D , Vincerzevskiene I , Shkolnikov VM . Education, survival, and avoidable deaths in Lithuanian cancer patients, 2001–2009. Acta Oncol. 2016;55:859‐864. doi:10.3109/0284186X.2016.1156739 27070947

[42] Vincerževskiene I , Jasilionis D , Austys D , Stukas R , Kaceniene A , Smailyte G . Education predicts cervical cancer survival: a Lithuanian cohort study. Eur J Public Health. 2017;27:421‐424. doi:10.1093/EURPUB/CKW261 28115421

[43] Shahrabi Farahani F , Paapsi K , Innos K . The impact of sociodemographic factors on the utilization of radiation therapy in breast cancer patients in Estonia: a register‐based study. Int J Equity Health. 2021;20:152. doi:10.1186/s12939-021-01497-0 34193144 PMC8247084

[44] Reile R , Veideman T . Health Behaviour among Estonian Adult Population, 2022. National Institute for Health Development; 2023.

[45] Hallik R , Innos K , Jänes J , Jõers K , Ratnik K , Veerus P . HPV self‐sampling in organized cervical cancer screening program: a randomized pilot study in Estonia in 2021. J Med Screen. 2025;32:19‐27. doi:10.1177/09691413241268819 39091000

