# Supplementary material for: Educational inequalities in cervical cancer mortality in the Baltic countries and Finland in the context of organized screening: A register‐based study 2000–2015
Source: Int J Cancer. 2026 Jan 24;158(12):3132–40. doi: 10.1002/ijc.70339 (PMC13106923; doi:10.1002/ijc.70339)
Supplement: Supplementary file 1 — Data S1: Supplementary Tables [file IJC-158-3132-s001.pdf]

Educational inequalities in cervical cancer mortality in the Baltic countries and Finland in the context of organized screening: a register-based study 2000–2015

Oskar Nõmm, Kaire Innos, Domantas Jasilionis, Juris Krumins, Pekka Martikainen, Kersti Pärna, Andrew Stickley, Mall Leinsalu

Supplementary materials:

Supplementary Tables 1–4

Supplementary Table 1 Age-standardized cervical cancer mortality among women aged 30–64 years in Finland, Estonia, Latvia and Lithuania, 2000–2015.

		Total	Age group 30–49	Age group 50–64
Country	Period	ASMR (95% CI)	ASMR (95% CI)	ASMR (95% CI)
Finland	2000–2007	1.6 (1.3–1.8)	1.1 (0.9–1.4)	2.3 (1.8–2.8)
	2008–2015	1.6 (1.3–1.8)	1.4 (1.1–1.7)	1.9 (1.5–2.3)
	Change	0.0	0.3	-0.4
Estonia	2000–2007	8.9 (7.8–10.2)	6.4 (5.2–7.9)	12.8 (10.7–15.2)
	2008–2015	9.6 (8.4–10.8)	5.1 (4.0–6.5)	16.5 (14.2–19.1)
	Change	0.7	-1.3	3.7
Latvia	2000–2007	9.8 (8.8–10.8)	7.3 (6.2–8.4)	13.6 (11.9–15.5)
	2008–2015	11.2 (10.2–12.3)	8.7 (7.5–10.0)	15.1 (13.3–17.0)
	Change	1.4	1.4	1.5
Lithuania	2001–2007	14.5 (13.5–15.6)	11.0 (9.9–12.1)	20.1 (18.2–22.1)
	2008–2015	13.3 (12.4–14.2)	9.9 (8.9–11.0)	18.5 (16.9–20.2)
	Change	-1.2	-1.1	-1.6

ASMR, age-standardized (European) mortality rate per 100 000 person years; CI, confidence interval; Change is calculated in comparison with the first period.

Supplementary Table 2 Age-standardized mortality rates and mortality rate ratios for cervical cancer among women aged 30–49 years, 2000–2015.

		ASMR (95% CI)		Change	Rate ratio (95% CI)	
Country	Educational level	2000–2007	2008–2015	ASMR	2000–2007	2008–2015
Finland	High	0.6 (0.3–1.0)	0.6 (0.4–1.0)	0.0	1	1
	Middle	1.0 (0.7–1.5)	1.9 (1.4–2.6)	0.9	1.70 (0.85–3.54)	3.03 (1.65–5.78)
	Low	2.5 (1.6–3.8)	2.2 (1.2–3.7)	-0.3	4.21 (2.10–8.86)	3.51 (1.57–7.68)
	Diff.	1.9	1.6			
Estonia	High	3.1 (1.9–4.8)	2.3 (1.3–3.8)	-0.8	1	1
	Middle	6.7 (5.0–8.8)	5.7 (4.1–7.8)	-1.0	2.15 (1.25–3.81)	2.49 (1.33–4.88)
	Low	24.0 (15.5–35.5)	21.0 (12.4–32.9)	-3.0	7.73 (4.10–14.73)	9.11 (4.28–19.32)
	Diff.	20.9	18.7			
Latvia	High	3.2 (1.9–5.1)	3.3 (2.1–5.1)	0.1	1	1
	Middle	7.9 (6.7–9.4)	9.3 (7.7–11.0)	1.4	2.50 (1.51–4.35)	2.78 (1.76–4.60)
	Low	13.1 (8.3–19.7)	24.1 (16.7–33.6)	11.0	4.13 (2.12–8.13)	7.23 (4.10–12.94)
	Diff.	10.0	20.8			
Lithuania	High	3.8 (2.6–5.4)	3.6 (2.5–5.0)	-0.2	1	1
	Middle	11.9 (10.6–13.3)	11.1 (9.8–12.6)	-0.8	3.13 (2.16–4.66)	3.09 (2.19–4.50)
	Low	30.5 (22.7–40.1)	24.6 (18.2–32.4)	-5.9	8.01 (5.03–12.88)	6.85 (4.37–10.76)
	Diff.	26.7	21.0			

ASMR, age-standardized (European) mortality rate per 100 000 person years; CI, confidence interval; Diff., ASMR difference between low- and highly educated. Change is calculated in comparison with the first period.

Supplementary Table 3 Age-standardized mortality rates and mortality rate ratios for cervical cancer among women aged 50–64 years, 2000–2015.

Country	Educational level		ASMR (95% CI) 2000–2007	2008–2015	Change ASMR	Rate ratio (95% CI) 2000–2007	2008–2015
Finland	High		1.4 (0.8–2.4)	1.3 (0.8–2.0)	-0.1	1	1
	Middle		1.8 (1.2–2.7)	1.7 (1.2–2.4)	-0.1	1.28 (0.66–2.55)	1.30 (0.72–2.37)
	Low		3.1 (2.3–4.1)	2.9 (1.9–4.1)	-0.2	2.16 (1.20–4.09)	2.18 (1.20–4.05)
		Diff.	1.7	1.6			
Estonia	High		7.1 (4.6–10.4)	9.8 (7.2–13.1)	2.7	1	1
	Middle		14.5 (11.2–18.4)	19.3 (15.7–23.4)	4.8	2.05 (1.28–3.40)	1.96 (1.37–2.85)
	Low		20.4 (14.3–28.3)	30.0 (20.4–42.6)	9.6	2.90 (1.68–5.03)	3.05 (1.87–4.92)
		Diff.	13.4	20.1			
Latvia	High		7.8 (5.0–11.6)	7.6 (5.2–10.6)	-0.2	1	1
	Middle		13.6 (11.3–16.1)	16.2 (13.9–18.8)	2.6	1.74 (1.12–2.81)	2.14 (1.47–3.19)
	Low		18.5 (14.1–23.8)	31.3 (22.2–42.8)	12.8	2.36 (1.45–3.97)	4.14 (2.54–6.75)
		Diff.	10.7	23.7			
Lithuania	High		8.9 (6.2–12.2)	7.0 (5.1–9.3)	-1.9	1	1
	Middle		20.4 (17.9–23.0)	20.0 (17.9–22.2)	-0.4	2.30 (1.62–3.34)	2.87 (2.10–4.01)
	Low		29.2 (23.7–35.7)	43.9 (34.4–55.1)	14.7	3.30 (2.23–4.96)	6.32 (4.30–9.36)
		Diff.	20.4	36.9			

ASMR, age-standardized (European) mortality rate per 100 000 person years; CI, confidence interval; Diff., ASMR difference between low- and highly educated. Change is calculated in comparison with the first period.

Supplementary Table 4 Impact of excluding register-only-based records on cervical cancer mortality among 30–64-year-old women in Latvia.

Period	Census + registry ASMR (95% CI)	Census ASMR (95% CI)	*P* value	Census + registry RR (95% CI)	Census RR (95% CI)
2000–2007	10.1 (9.2–11.1)	9.8 (8.9–10.8)	0.653	1	1
2008–2015	12.3 (11.2–13.4)	11.7 (10.6–12.8)	0.435	1.21 (1.07–1.38)	1.19 (1.04–1.36)

ASMR, age-standardized (European) mortality rate per 100 000 person years; CI, confidence interval.
RR, rate ratios comparing the 2008–2015 period with the 2000–2007 period (reference category).
P values are for the ASMR differences resulting from excluding register-only-based records.
